# Journal subscription expenditure of UK higher education institutions

**DOI:** 10.12688/f1000research.5706.3

**Published:** 2015-07-06

**Authors:** Stuart Lawson, Ben Meghreblian

**Affiliations:** 1 Independent researcher, Brighton, East Sussex, BN2 5PF, UK; 2 Independent researcher, East Twickenham, Middlesex, TW1 2BX, UK

**Keywords:** Journals, subscription, higher education institution

## Abstract

The academic libraries of higher education institutions (HEIs) pay significant amounts of money each year for access to academic journals. The amounts paid are often not transparent especially when it comes to knowing how much is paid to specific publishers. Therefore data on journal subscription expenditure were obtained for UK HEIs using a series of Freedom of Information requests. Data were obtained for 153 HEIs’ expenditure with ten publishers over a five-year period. The majority of institutions have provided figures but some are still outstanding. The data will be of interest to those who wish to understand the economics of scholarly communication and see the scale of payments flowing within the system. Further research could replicate the data collection in other jurisdictions.

## Introduction

The amount of money paid by higher education institutions (HEIs) to access academic journals is of high interest to the academic community, and academic libraries in particular as they are responsible for the vast majority of journal purchases. In light of current trends within academic publishing towards open access models rather than subscription models, the economics of the publishing industry have come under increasing scrutiny, but accurate data about the flow of money within the system is difficult to come by. Libraries do not usually publish details of their expenditure with individual publishers and there is no official source of these data. This situation led to undertaking this research to make journal subscription expenditure openly available.

Freedom of Information (FOI) requests were sent to HEIs to obtain the data. While the authors considered using a diplomatic approach and asking individual libraries to publish their data, this would have taken a considerable amount of time, and while some libraries may have been happy to publish the data themselves, others may not have seen the value in it. The situation is also complicated by the fact that some publishers insist on having non-disclosure clauses in their contracts with libraries, which prohibit them from disclosing some aspects of the deals. The UK’s Freedom of Information Act (2000) overrides these clauses and allows full data to be obtained by sending FOI requests.

It is hoped that the data contained within this dataset will contribute to a better informed discussion surrounding the issue of how scholarly communication could or should be funded. Further research could undertake a similar endeavour in the 100 other countries ( McIntosh, 2014) which have FOI laws, in order to work towards understanding the costs of scholarly communication on a global scale.

## Materials and methods

A list of HEIs was created based on UK institutions which the Higher Education Statistics Agency ( HESA, n.d.) collects data about. In order to obtain data which cover the majority of HEI journal expenditure, ten of the largest publishers of academic journals were chosen (Elsevier, Wiley, Springer, Taylor & Francis, Sage, Nature Publishing Group, Oxford University Press, Cambridge University Press, Institute of Physics Publishing, and Royal Society of Chemistry).

Each institution was then sent four separate FOI requests via the website whatdotheyknow.com, which sends FOI requests on behalf of UK citizens. The site was chosen because it places all correspondence in the public domain indefinitely, thus ensuring that the data will be verifiable. The four requests were grouped as follows: Group 1 - Wiley, Springer, OUP; Group 2 - Taylor & Francis, Sage, CUP; Group 3 - Elsevier; Group 4 - Nature Publishing Group, RSC, IOP. The groupings were chosen to ensure that each request would not be too onerous for an HEI to respond to, as stipulated under the UK’s FOI law. Elsevier data were requested separately because the nature of their contract with libraries means that the institution must contact Elsevier when it receives a request, thus increasing the time burden on institutions. An individual known to the authors sent similar requests to Russell Group universities for Wiley, Springer, and OUP expenditure earlier in 2014, so these requests were not duplicated and the data obtained by them ( Brook, 2014) have now been incorporated into the main dataset.

The figures should include payments made directly to the publishers as well as any payments made to subscription agents or intermediaries for the purchase of, and/or access to, the publishers' academic journals. Institutions were asked to provide data for the payment for journal packages such as Jisc Collections’ NESLi agreement, as well as for individual journals, and to include VAT where possible. Since the authors are relying solely on data provided by the HEIs it is not possible to independently verify whether all of these aspects of the requests have been adhered to. While this may result in some inaccuracies in individual figures, the authors do not consider that the overall scale will be unduly affected.

Data were requested for five calendar years (2010–14). Some institutions provided data in financial years, which for UK academic institutions is from August-July. In these cases the financial year was mapped on to the second of the two years, for example 2009–10 was mapped on to 2010. This is because although during the financial year 2009–10 it is possible that the money was actually transferred during 2009, it will have been used to pay for subscriptions for 2010. Amounts paid in currencies other than GBP have been converted into GBP based on the exchange rate on 1 January of the year in question. Most figures included VAT, and although in early versions of the dataset VAT was added to those figures which excluded it (at UK rates of 17.5% in 2010 and 20% in 2011–14), this is no longer the case. In the UK, VAT is only applied to electronic and not print publications. Since it is not usually clear what proportion of the expenditure is on print and what is on electronic subscriptions, VAT has not been added to the figures. The resultant figures are therefore slightly lower than they should be but it was felt that this is preferable to the risk of unduly inflating them.

Caution must be exercised when comparing the amount that an institution pays to the amount paid by other institutions, because it is likely that they are not purchasing access to exactly the same ‘package’ of content. In some cases institutions pay for large bundles of titles, and in other cases they pay for individual titles. We did not ask institutions to provide precise details of what they purchased because we believed that doing so could add significantly to the time it would take for them to produce responses to the requests, which may well have led to refusals. A few institutions did provide this level of detail in their response.

The dataset is now well-populated with over £430m of expenditure but it is still incomplete because at the time of writing, out of the 589 FOI requests that were sent there are still 28 outstanding for which data has not yet been provided. Further data will be incorporated into the dataset as it becomes available.

## Data availability

Data can be accessed directly via Figshare at http://figshare.com/articles/Journal_subscription_costs_FOIs_to_UK_universities/1186832, http://dx.doi.org/10.6084/m9.figshare.1186832 ( Lawson & Meghreblian, 2014).

Data were obtained from each institution sending separate FOI requests via the website whatdotheyknow.com. Requests can be viewed individually at https://www.whatdotheyknow.com/user/stuart_lawson#foi_requests and https://www.whatdotheyknow.com/user/ben_meghreblian#foi_requests.
