# Supplementary material for: Journal subscription expenditure of UK higher education institutions
Source: F1000Res. 2015 Jul 6;3:274. Originally published 2014 Nov 13. [Version 3] doi: 10.12688/f1000research.5706.3 (PMC4304214; doi:10.12688/f1000research.5706.3)
Supplement: Peer review history

# Peer review: Referee response for version 3

**DOI:** 10.5256/f1000research.7255.r9342

**Published:** 2015-07-08

**Authors:** John Dupuis

**Affiliations:** 1 Steacie Science & Engineering Library, York University, Toronto, ON, Canada

I approve of the current revisions to this article.

I have read this submission. I believe that I have an appropriate level of expertise to confirm that it is of an acceptable scientific standard.

# Peer review: Referee response for version 2

**DOI:** 10.5256/f1000research.6300.r7247

**Published:** 2015-01-20

**Authors:** Jingfeng Xia

**Affiliations:** 1 Department of Informatics and Computing, Indiana University, Indianapolis, IN, USA

Data sharing is critical for decreasing duplicated work and increasing the possibility of comparative analyses. Researchers and practitioners will be able to develop diverse thinking and design appropriate collaborations based upon existing data. Data sharing will also allow others to validate one another's discoveries so as to encourage research accountability and transparency.

Stuart Lawson and Ben Meghreblian made a great effort to open their data on the subject of journal subscription expenditure. Their research design for collecting such data looks sound. People can expect to examine their data and make possible comparisons across libraries.

While using their data, one needs to be aware that journal subscriptions are very complicated in library practices. Many variables may potentially affect the pricing and make comparisons tricky. Furthermore, their published data are aggregated, preventing users from drilling down through detailed numbers. However, it is a good start. We look forward to reading more data published for free access.

I have read this submission. I believe that I have an appropriate level of expertise to confirm that it is of an acceptable scientific standard.

# Peer review: Referee response for version 2

**DOI:** 10.5256/f1000research.6300.r7246

**Published:** 2015-01-20

**Authors:** John Dupuis

**Affiliations:** 1 Steacie Science & Engineering Library, York University, Toronto, ON, Canada

The authors have done an excellent job of gathering and presenting data about journal subscription expenditures in the UK. I have no major suggestions for the current version of the article.

The only minor suggestion I would have is that the authors may have wanted to include more information and links to similar data that has already been collected for both the UK and other countries, such as the data collected by Timothy Gowers or Theodore C. Bergstrom *et al.*

I have read this submission. I believe that I have an appropriate level of expertise to confirm that it is of an acceptable scientific standard.

# Peer review: Referee response for version 2

**DOI:** 10.5256/f1000research.6300.r7291

**Published:** 2015-01-13

**Authors:** Subbiah Arunachalam

**Affiliations:** 1 Centre for Internet and Society, Bangalore, India

Academic and research libraries purchase large number of professional journals from publishers, often in packages (also referred to as bundles). Often publishers insist on non-disclosure clauses in the contracts they sign with subscribing institutions, and they sell journals to different institutions at different prices. Clever negotiators (as those in the University of California system) bargain hard and obtain better deals, but others pay needlessly high prices. Selling the same product at different prices to different consumers is unethical. Till recently it was very difficult or even impossible to obtain information on how much a university pays a (commercial) publisher as subscription to journals. Now people like Lawson and Meghreblian (and Bergstrom, Timothy Gowers) have found a way to overcome this unethical practice of publishers. 

This short paper and the data is on how much each university in the UK pays seven publishers by way of subscription to journals. The paper is well written and it makes public the voluminous data they have collected using the Freedom of Information Act.

I have read this submission. I believe that I have an appropriate level of expertise to confirm that it is of an acceptable scientific standard.

# Peer review: Referee response for version 1

**DOI:** 10.5256/f1000research.6101.r6694

**Published:** 2014-11-14

**Authors:** Theodore Bergstrom

**Affiliations:** 1 Economics Department, University of California Santa Barbara, Santa Barbara, CA, USA

The authors have collected a set of data that  is likely to be very useful both to researchers who investigate journal pricing and to librarians who want to know more about what others are paying when they make their own bargains with publishers.   The methods of collection seem sound and the work appears to be carefully done.

There is one thing that I would like to see clarified.  The report states the amounts that are paid to each publisher, but do not make it clear whether all of the reported institutions are buying the same thing from the publishers.   For example, Elsevier markets a "Freedom package" which includes a published list of journals constituting most, but not all, of the journals they publish.

They also market a "Complete package" which does not include the entire Freedom package, but basically a set of journals to which the university has previously subscribed.  Elsevier also separately markets the Cell Press journals, a "clinical medicine package",  a series of monographs called "advances in ...." Some of the contract totals that I have seen include all or some of these.

Similarly, Springer sells not only its journal package, but also a large number of textbooks and monographs.  Some libraries' total expenditure with Springer may include these.  It would be good to know if your totals are just for the journal bundle.

With some of the other publishers, it also is not entirely clear whether the totals reported are from libraries that do not subscribe to the publisher's entire journal bundle but only to some limited subset.  Once again, it would be good to have this clarified.

I have read this submission. I believe that I have an appropriate level of expertise to confirm that it is of an acceptable scientific standard.

# Response

**Published:** 2014-11-17

**Authors:** Stuart Lawson

Thank you for your comments, you're right that it would be best to clarify what is actually being purchased in each instance, as far as we are able. When one institution pays a different amount to another institution, it is likely that they are not purchasing access to exactly the same 'package' of content. We did not ask institutions to provide this information as part of the FOI requests because we believed that in some cases it would add significantly to the time it would take for them to produce responses, which may have led to refusals. Some institutions did provide this level of details in their response; those who purchase access directly from a publisher rather than through Swets appear to find it easier to provide such data.

One way we could highlight this would be to mark the figures in some way to indicate whether an institution has paid for the complete package of content from a given publisher. However, in order to do this consistently, we would need to contact most or all of the institutions again in order to confirm this with them. For some institutions this information can be gleaned from the original FOI response which is viewable on whatdotheyknow.com.

The most accurate way to compare between different institutions would be if we asked for each institution to release the title list of purchased content for each publisher during each year. Through a project I am working on at Jisc Collections, which aims to build an 'entitlement registry' of this information for all UK institutions, I have learned that it may be very difficult for all institutions to provide that data.
